# Retraction: The effect of a sports chiropractic manual therapy intervention on the prevention of back pain, hamstring and lower limb injuries in semi-elite Australian Rules footballers: A randomized controlled trial

**DOI:** 10.1186/1471-2474-12-200

**Published:** 2011-09-13

**Authors:** Wayne Hoskins, Henry Pollard

**Affiliations:** 1 Department of Chiropractic, Macquarie University, Sydney, NSW 2109, Australia

## 

The journal has been informed by the authors' institution that, contrary to the statement in this article [1], the Macquarie University Human Ethics Committee did not receive an application for ethics approval for this study. As the study was conducted without institutional ethics committee oversight, this article has been retracted.

## Pre-publication history

The pre-publication history for this paper can be accessed here:

http://www.biomedcentral.com/1471-2474/12/200/prepub
